# From Bad to Disaster: Iatrogenic Fracture Followed by Knee Perforation With Bone Fragments During Femoral Nailing

**DOI:** 10.7759/cureus.21686

**Published:** 2022-01-28

**Authors:** Povilas Masionis, Narūnas Porvaneckas, Valentinas Uvarovas, Igoris Šatkauskas, Tomas Sveikata, Giedrius Kvederas

**Affiliations:** 1 Orthopedics, Vilnius University, Faculty of Medicine, Institute of Clinical Medicine, Clinic of Rheumatology, Orthopaedic Traumatology and Reconstructive Surgery, Centre of Orthopedics and Traumatology, Republican Vilnius University Hospital, Vilnius, LTU; 2 Vilnius University, Faculty of Medicine, Institute of Clinical Medicine, Clinic of Rheumatology, Orthopaedic Traumatology and Reconstructive Surgery, Centre of Orthopedics and Traumatology, Republican Vilnius University Hospital, Vilnius, LTU; 3 Centre of Orthopedics and Traumatology, Vilnius University, Faculty of Medicine, Institute of Clinical Medicine, Clinic of Rheumatology, Orthopaedic Traumatology and Reconstructive Surgery, Vilnius University Hospital, Santaros Clinics, Vilnius, LTU

**Keywords:** fracture, knee arthroplasty, arthrodesis, nailing, nail

## Abstract

Intramedullary nailing is the preferred treatment method in tibial, femoral shaft, and sub- or intertrochanteric fractures. Despite good results, a number of complications have been well-characterized. Joint perforation by bone fragment during nail insertion is one such complication.

We report an eventful case of a 63-year-old female patient who presented with an intertrochanteric fracture. Osteosynthesis with a short proximal femoral nail was complicated by a well-known and recognized on-time complication - an iatrogenic fracture of the femoral shaft. However, it was further complicated by knee arthrodesis by a bone fragment when a long nail was inserted. The bone fragment not only “closed” the knee joint, but fractured the medial tibial condyle and protruded into the medial soft tissues of the joint. This misfortune was not recognized intraoperatively and led to revision surgery in the short term and joint post-traumatic arthrosis with resultant total knee replacement in the long term.

## Introduction

Over the past decade, intramedullary nailing (IMN) has become the preferred treatment method in tibial, femoral shaft, and sub- or intertrochanteric fractures [1-4]. Despite good results, a number of complications are described: rotational and axial malalignment, shortening of the limb, intraoperative fracture, hardware breakage, joint penetration by guidewire or nail, malunion, nonunion, infection, and neurovascular damage [5-9]. Joint perforation by bone fragment during nail insertion is an extremely unique complication. At the time of writing, a PubMed database search using the terms complication AND nail AND fragment AND bone AND joint revealed five cases: three cases of femoral bone fragment penetration to the knee joint, one case of tibial fragment penetration to the ankle joint, and one case of ankle joint perforation by bone fragment [10-12]. To our knowledge, it is the first described case of knee joint arthrodesis and complete tibial condyle perforation by femoral bone fragment during nailing of an intertrochanteric fracture, which led to posttraumatic arthrosis and total knee arthroplasty.

## Case presentation

**Figure 1:**
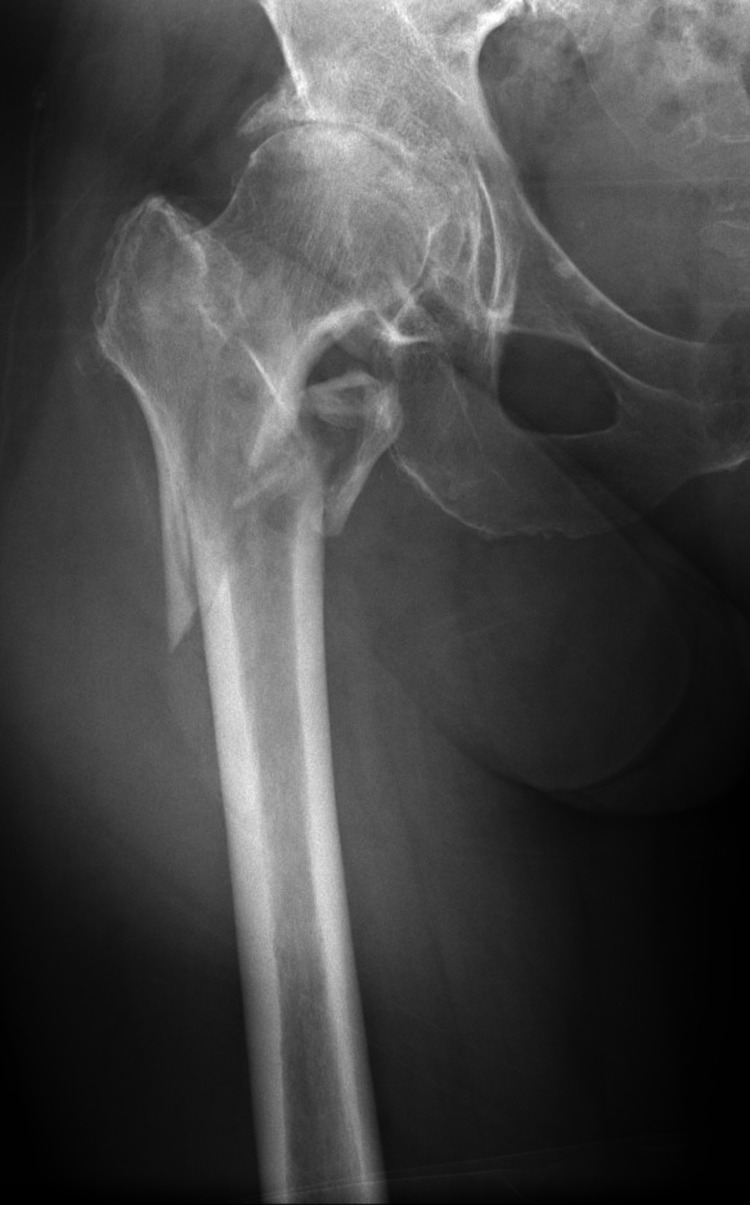
Orthopaedic Trauma Association 31 A3.3 fracture of the right proximal femur

A 63-year-old woman sustained an intertrochanteric fracture (Orthopaedic Trauma Association 31 A3.3) after falling from her standing height (Figure 1).

**Figure 2:**
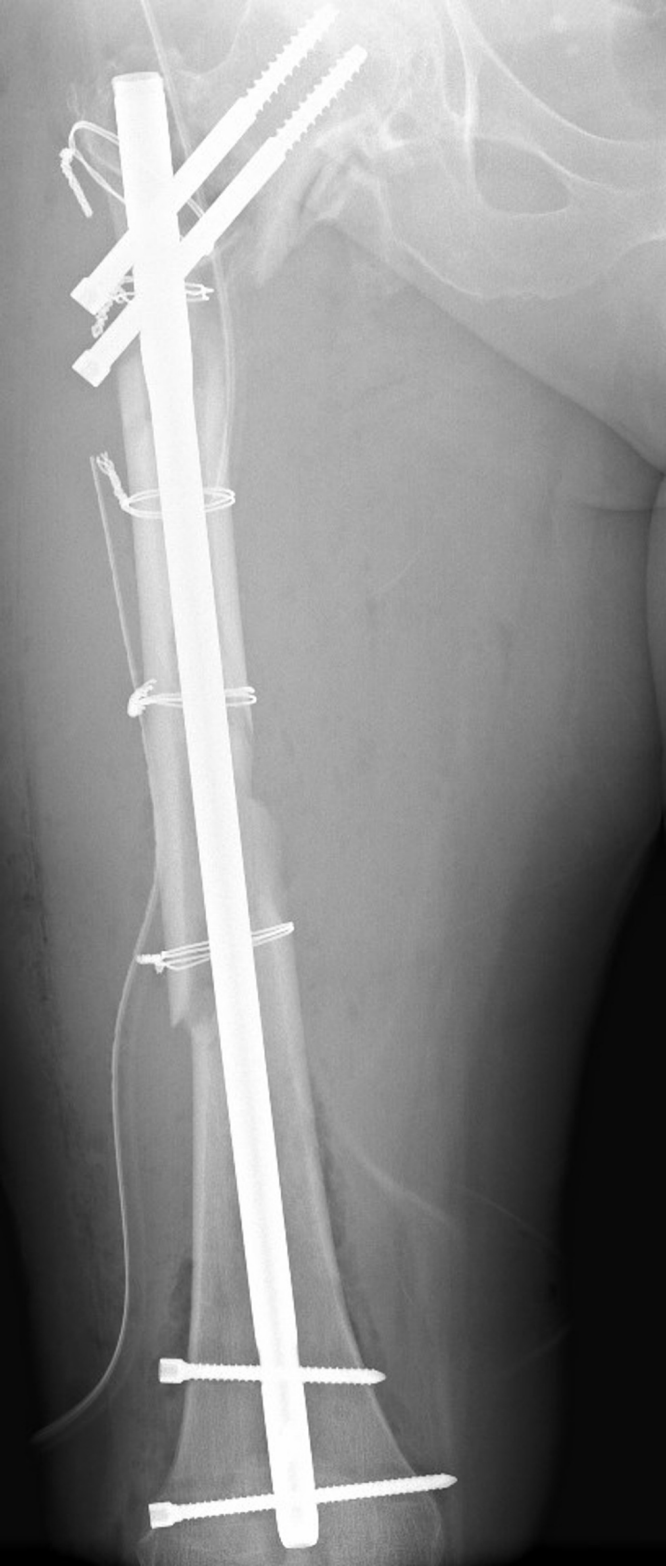
Postoperative X-ray of intramedullary osteosynthesis with a long nail

Ipsilateral coxarthrosis was present, but it was decided to heal the fracture before joint arthroplasty. On the following day, the patient underwent closed reduction internal fixation surgery with a proximal femoral nail (PFN) on the fracture table. However, the introduction of the 12x240 mm PFN was complicated by a spiral fragmented fracture of the femoral shaft. The nail was removed, the fracture reduced in an open fashion, and held by four wire cerclages. An 11x380 mm Gama nail was used for definitive fixation; the surgery took six hours and 10 minutes (Figure 2).

**Figure 3:**
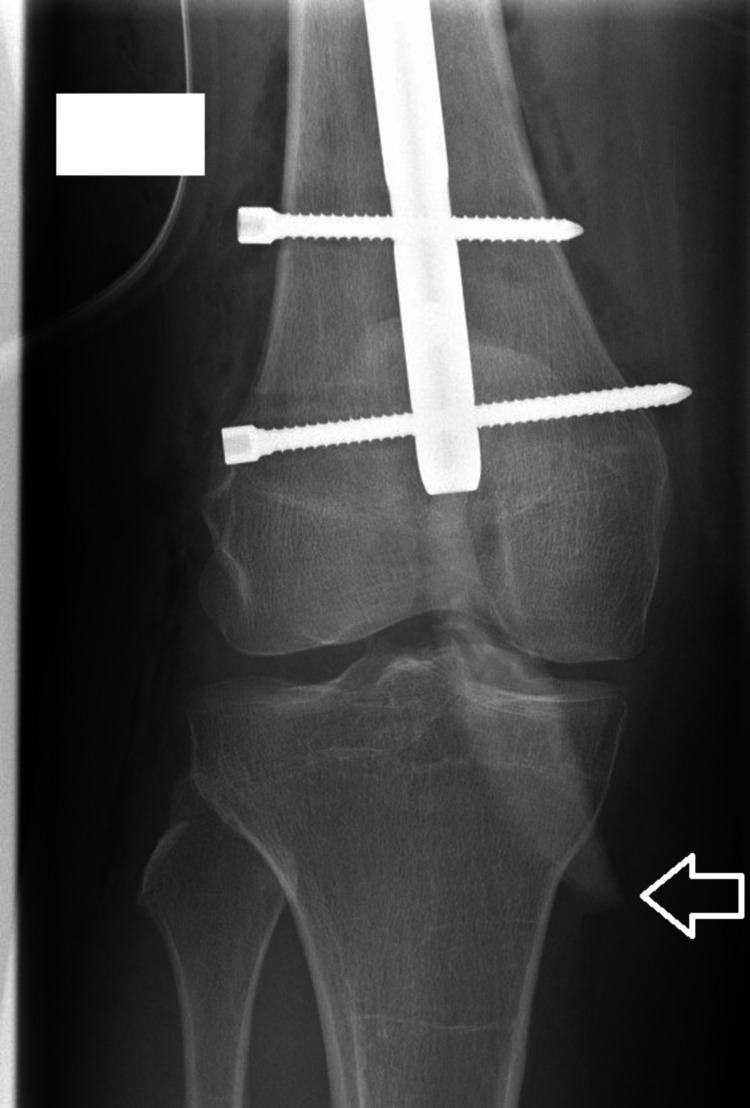
White arrow marks the intraarticular bone fragment on an anteroposterior postoperative X-ray

**Figure 4:**
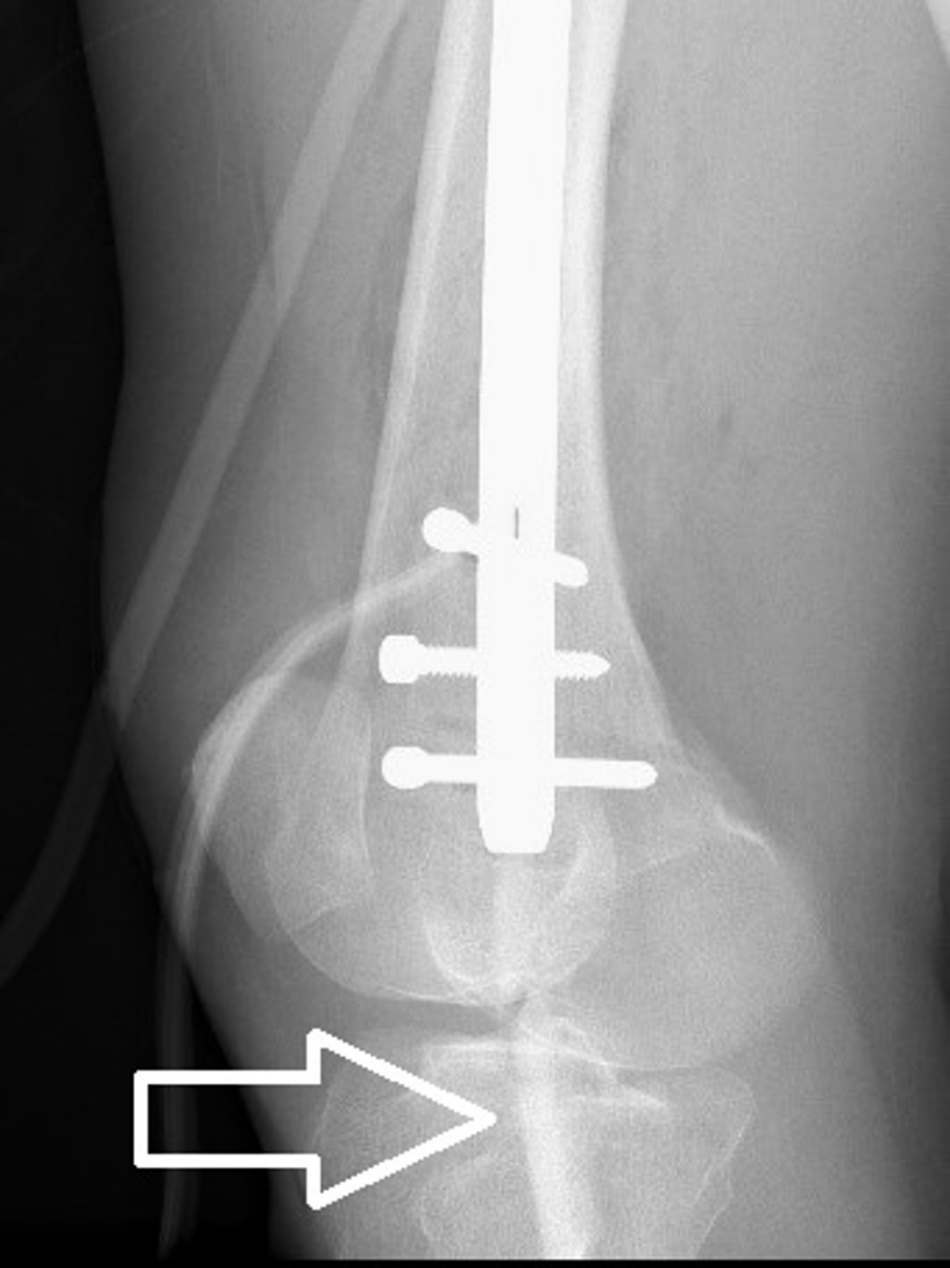
White arrow marks intraarticular bone fragment in lateral postoperative x-rays.

Postoperatively, the patient complained about persistent right knee pain that was not reduced by opioids. Furthermore, she was not able to bear weight or ambulate; her knee was stiff, with no movements. Careful examination of postoperative X-rays revealed a 60 x 10 mm bony fragment starting from the distal tip of the nail, going through knee joint space into the medial tibial condyle and perforating its medial cortex (Figures 3-4). 

**Figure 5:**
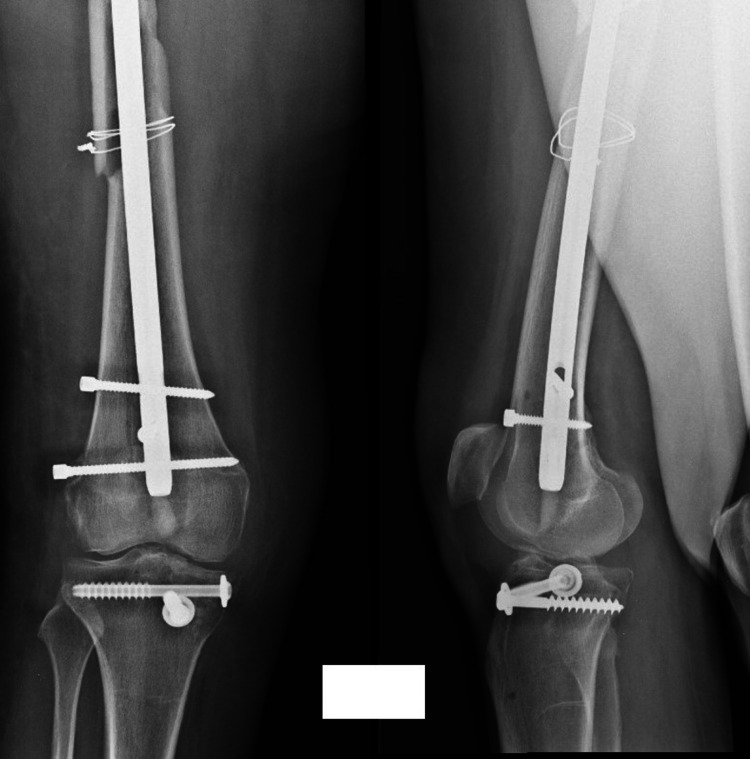
The bone fragment was removed through the medial parapatellar approach - the medial tibial condyle fracture was fixed by two lag screws

An immediate CT scan confirmed the diagnosis of knee joint penetration by the femoral fracture fragment with the fracture of the medial tibial condyle. Ten days after the first surgery, the fragment was removed through the medial parapatellar approach and the medial tibial condyle was fixed by lag screws (Figure 5).

The further hospital stay was uneventful; the patient was ambulated and the knee pain resolved.

Because we focus on the course of the right knee pathology, additional surgery of the right hip is mentioned without technical details. After a further half-year, the femoral shaft healed, although the patient had to undergo total hip arthroplasty (THA) because of intertrochanteric fracture non-union with cut out of the screws from the femoral head and previous arthrosis.

**Figure 6:**
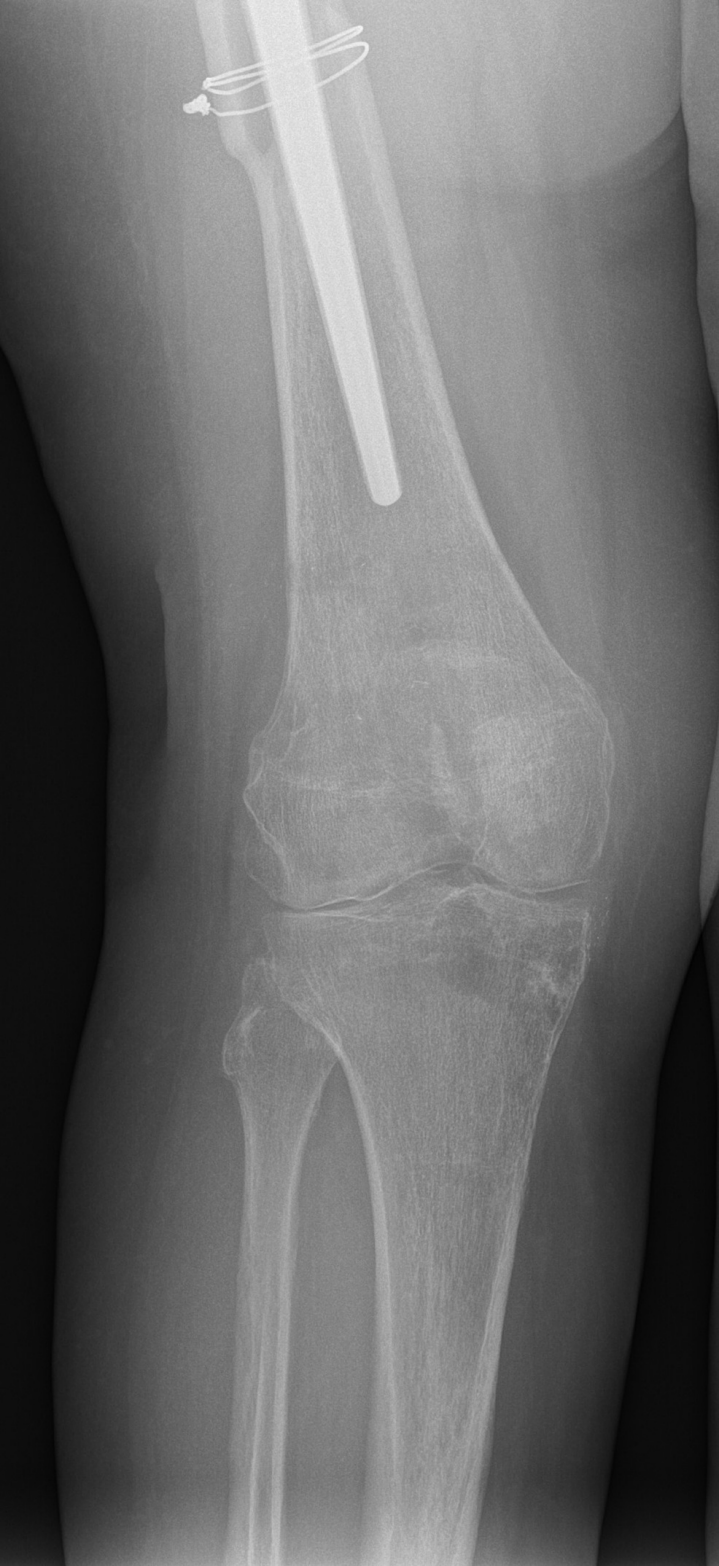
Two years after the trauma, posttraumatic gonarthrosis is present

Two years after the trauma, the patient complained about right knee pain and stiffness. Clinical evaluation revealed osteoarthritis of the right knee joint with a range of motion (ROM) of ext/0/flex 0/5/5. Clinical and laboratory workup showed no signs of infection: the white blood cell count was normal, erythrocyte sedimentation rate (ESR) 7 mm/H, c-reactive protein (CRP) 2 mg/l. The X-rays were consistent with posttraumatic gonarthrosis diagnosis (Figure 6). 

**Figure 7:**
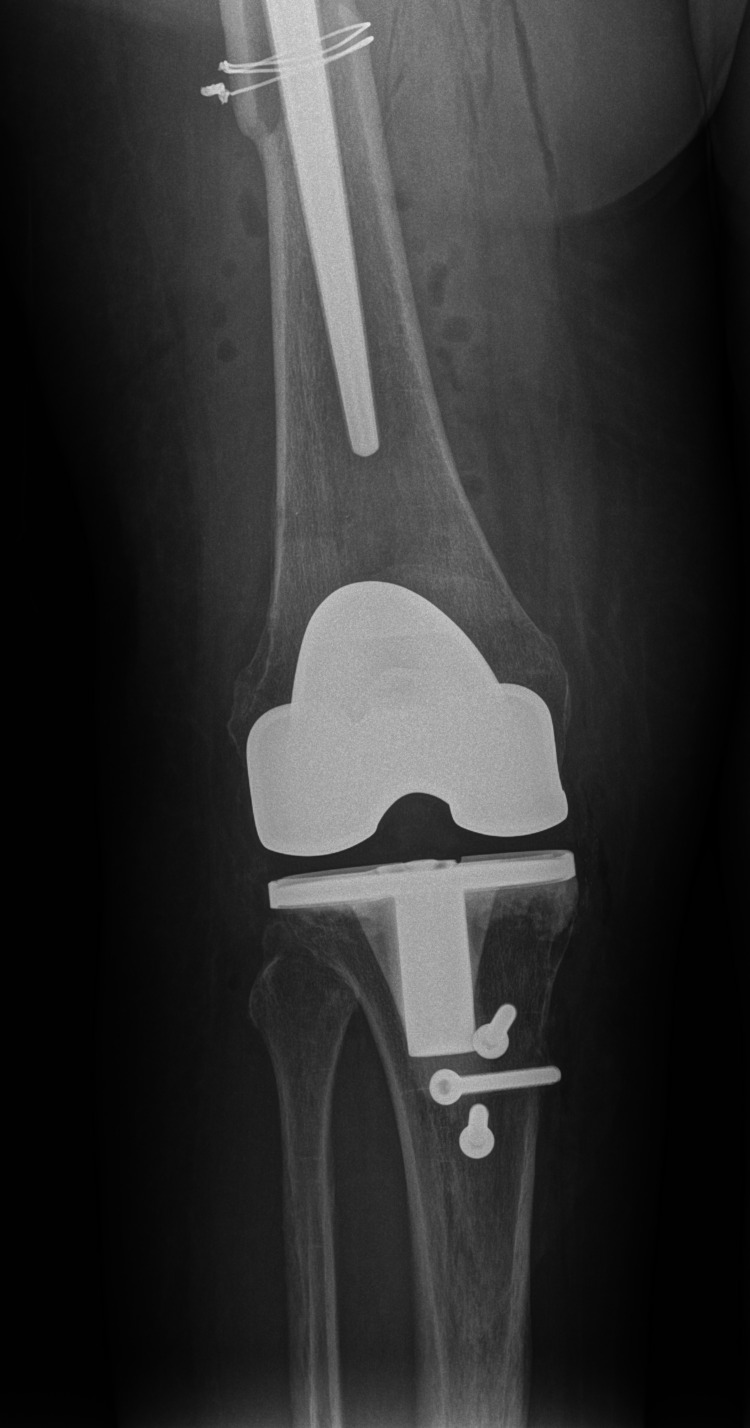
Anteroposterior X-ray of TKA and tibial tuberosity fixation TKA: total knee arthroplasty

**Figure 8:**
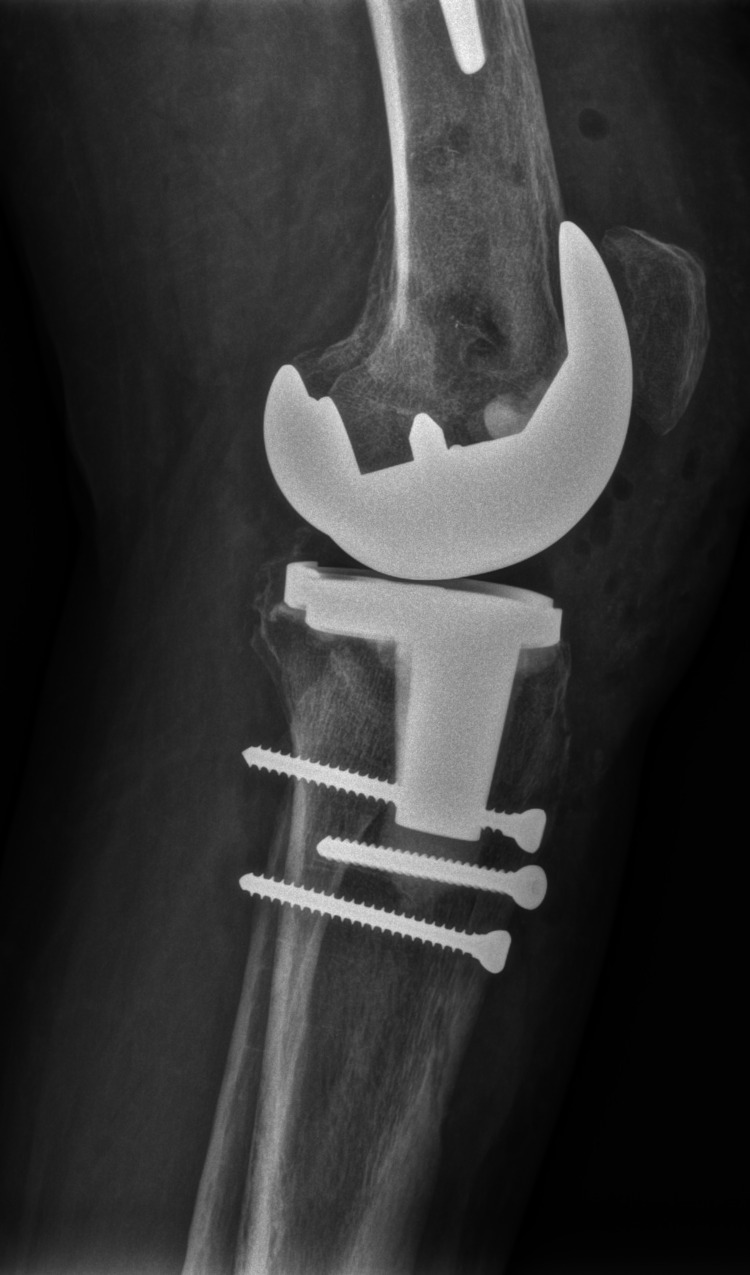
Lateral X-ray of TKA and tibial tuberosity fixation TKA: total knee arthroplasty

The patient was scheduled for total knee arthroplasty (TKA). The knee joint was opened through the medial parapatellar approach. Excessive intraarticular scar tissue was found. Despite all the adhesions being removed, it was impossible to flex the knee. The decision was made to use the extensile approach with osteotomy of the tibial tuberosity. A defect of 30 percent of the size of the medial tibial condyle was present in its central part. After removal of both cruciate ligaments, a satisfactory ROM of 0/0/80 was reached with NexGen LPS (Zimmer Biomet, Warsaw, Indiana) posterior stabilized total knee prosthesis (Figures 7-8).

Five years after the initial trauma, the patient is walking with a stick and has complaints of mild pain in the area of the right greater trochanter. She is able to walk without any aid but because of the positive Trendelenburg test, limping makes gait difficult. There are no signs of infection. ROM of the right knee is ext/0/flex 0/5/45. Despite the difficult path, she is taking care of her livestock and plant farm without any assistance.

## Discussion

To our knowledge, this is the first case report of not only complete knee joint perforation but also perforation of the medial tibial condyle into the medial soft tissue of the knee by a bone fragment. Joint perforation by bone fragment during IMN is not only extremely unique complication but difficult to diagnose too. Delayed diagnosis was present in two of five cases described in the literature: knee penetration after the subtrochanteric fracture INM was diagnosed 28 days after the trauma and ankle joint arthrodesis after tibial INM 11 weeks after the surgery [11]. In the first case, the knee pain and difficulty to ambulate by the patient were blamed on the spine injury, and in the second case, the ankle joint pain was considered as a normal occurrence after the surgery. However, both cases shared the same problem - control X-rays did not involve the joint distal to the nail. To continue, in two of five cases, joint penetration was detected in postoperative X-rays, and only in one case, a complication was detected before the end of the surgery [10,12]. In our case, the knee joint was involved in control films, but they were interpreted as normal and only extreme pain of the knee forced the physician to re-evaluate the X-rays. These findings emphasize the importance of a quality radiological examination before termination of surgery - an exploration of the adjacent are is mandatory not only to access the rotation and axis of the limb but also to investigate joint space too. Complications found before the termination of the surgery could be solved under the same anesthesia.

Furthermore, in our opinion, joint penetration by fracture fragment during IMN can be named as an implant-specific complication - all cases happened in cannulated nails. A “spindle” or “rhombus” shaped bone fragment settles by its sharp edge in the opening of the nail canal and acts as the arrow tip by the opposite sharp edge during nail insertion. A guidewire should prevent this from happening, although proximal migration and a wire of insufficient thickness are documented as prone to this complication [12].

Intraoperative fracture during IMN is a well-documented complication; it is devastating to surgeons not only in an emotional way but in a physical way too as surgery becomes prolonged and more extensive. A rise in stress and exhaustion has a great impact on a surgeon’s judgment and decision-making [13]. A safety protocol of having a second opinion of a “fresh” surgeon before ending the surgery should be considered in every hospital.

## Conclusions

In conclusion, we can state that joint penetration or perforation by bone fragment is an extremely rare and difficult-to-diagnose complication that could lead to posttraumatic arthrosis and total joint arthroplasty. Surgeons should be always aware of possible intraoperative complications, although it is impossible to know them all. Key to timely diagnosis is a careful examination of the adjacent joints of nailed bone under the C arm and the key to early diagnosis is a careful examination of correct length immediate postoperative X-rays. This complication is implant-specific to cannulated nails. In the event of a complication, the safety protocol of mandatory second intraoperative opinion should be considered to prevent further disasters.
